# Physical Exercise and Mitochondrial Disease: Insights From a Mouse Model

**DOI:** 10.3389/fneur.2019.00790

**Published:** 2019-07-25

**Authors:** Carmen Fiuza-Luces, Pedro L. Valenzuela, Sara Laine-Menéndez, Miguel Fernández-de la Torre, Verónica Bermejo-Gómez, Laura Rufián-Vázquez, Joaquín Arenas, Miguel A. Martín, Alejandro Lucia, María Morán

**Affiliations:** ^1^Mitochondrial and Neuromuscular Diseases Laboratory, Research Institute of Hospital 12 de Octubre (i+12), Madrid, Spain; ^2^Physiology Unit, Systems Biology Department, University of Alcalá, Madrid, Spain; ^3^Spanish Network for Biomedical Research in Rare Diseases (CIBERER), Madrid, Spain; ^4^Faculty of Sports Sciences, European University of Madrid, Madrid, Spain; ^5^Spanish Network for Biomedical Research in Fragility and Healthy Aging (CIBERFES), Madrid, Spain

**Keywords:** rare diseases, mitochondrial diseases, OXPHOS, harlequin mutant mouse, resistance training, AIF deficiency, respiratory chain complex I

## Abstract

**Purpose:** Mitochondrial diseases (MD) are among the most prevalent neuromuscular disorders. Unfortunately, no curative treatment is yet available. This study analyzed the effects of exercise training in an animal model of respiratory chain complex I deficiency, the Harlequin (*Hq*) mouse, which replicates the clinical features of this condition.

**Methods:** Male heterozygous Harlequin (*Hq*/Y) mice were assigned to an “exercise” (*n* = 10) or a “sedentary” control group (*n* = 11), with the former being submitted to an 8 week combined exercise training intervention (aerobic + resistance training performed five times/week). Aerobic fitness, grip strength, and balance were assessed at the beginning and at the end of the intervention period in all the *Hq* mice. Muscle biochemical analyses (with results expressed as percentage of reference data from age/sex-matched sedentary wild-type mice [*n* = 12]) were performed at the end of the aforementioned period for the assessment of major molecular signaling pathways involved in muscle anabolism (mTOR activation) and mitochondrial biogenesis (proliferator activated receptor gamma co-activator 1α [PGC-1α] levels), and enzyme activity and levels of respiratory chain complexes, and antioxidant enzyme levels.

**Results:** Exercise training resulted in significant improvements in aerobic fitness (−33 ± 13 m and 83 ± 43 m for the difference post- vs. pre-intervention in total distance covered in the treadmill tests in control and exercise group, respectively, *p* = 0.014) and muscle strength (2 ± 4 g vs. 17 ± 6 g for the difference post vs. pre-intervention, *p* = 0.037) compared to the control group. Higher levels of ribosomal protein S6 kinase beta-1 phosphorylated at threonine 389 (156 ± 30% vs. 249 ± 30%, *p* = 0.028) and PGC-1α (82 ± 7% vs. 126 ± 19% *p* = 0.032) were observed in the exercise-trained mice compared with the control group. A higher activity of respiratory chain complexes I (75 ± 4% vs. 95 ± 6%, *p* = 0.019), III (79 ± 5% vs. 97 ± 4%, *p* = 0.031), and V (77 ± 9% vs. 105 ± 9%, *p* = 0.024) was also found with exercise training. Exercised mice presented with lower catalase levels (204 ± 22% vs. 141 ± 23%, *p* = 0.036).

**Conclusion:** In a mouse model of MD, a training intervention combining aerobic and resistance exercise increased aerobic fitness and muscle strength, and mild improvements were found for activated signaling pathways involved in muscle mitochondrial biogenesis and anabolism, OXPHOS complex activity, and redox status in muscle tissue.

## Introduction

Mitochondrial diseases (MD) encompass a heterogeneous group of rare diseases caused by defects in oxidative phosphorylation (OXPHOS) (1, 2), and are among the most common neuromuscular disorders, but showing variable estimated prevalence (3–5). Given the ubiquity of mitochondria and their essential role in ATP production, MD can affect several tissues and those with higher metabolic demands, particularly (but not only) the skeletal muscle, are usually the most affected, with mitochondrial myopathy and poor exercise capacity (as reflected by low levels of aerobic fitness) being frequent features among patients with MD (6, 7).

Although no curative treatment is yet available for MD, preclinical evidence from animal and *in vitro* studies suggests that promoting the increase in mitochondrial content or mitochondrial biogenesis, through the activation of peroxisome proliferator-activated receptor gamma co-activator 1-alpha (PGC-1α) might be a therapeutic option (8, 9). In this respect, regular endurance exercise is known to be a potent stimulus for muscle mitochondrial biogenesis (10), and in fact previous research has shown that endurance-based exercise interventions can increase the muscle oxidative capacity of patients with MD, as directly reflected by increases in the activity of citrate synthase (CS) and respiratory chain complexes in skeletal muscle biopsies (11–13), or indirectly, by improvements in patients' aerobic fitness (11–18). Another exercise modality, resistance or “strength” training (e.g., weight lifting), is also known to promote muscle mitochondrial biogenesis (19) together with other benefits, notably improvements in muscle mass and strength (20), but scarce data are available in MD patients. One study assessed the effects of a 12 week resistance training program in 8 patients with mitochondrial DNA large scale deletions, showing strength improvements and a decrease in mitochondrial DNA heteroplasmy without side effects (21). Another study found an increase in upper-body muscle strength after 12 weeks of combined resistance and endurance training in 10 mitochondrial patients with biopsy diagnosis of MD but with no genetic confirmation (22). Finally, we recently described significant improvements in upper-, lower-body, and respiratory muscle strength in 12 well-characterized MD patients (all with genetic diagnosis) after 8 weeks of combined resistance, endurance and respiratory training (14).

On the other hand, studying mouse models of MD allows to gain mechanistic insight into the molecular processes underlying exercise benefits in these conditions. In this respect, although previous exercise training studies have been published with mouse models of respiratory chain complex IV deficiency (23) or of a defect in mitochondrial polymerase gamma (i.e., “mutator” mice) (24–26), the effects of exercise-training on a mouse model of the commonest mitochondrial defect, respiratory chain complex I deficiency, have yet not been assessed. Further, the effects of resistance training remain to be determined in mouse models of MD.

The aim of the present study was to determine the effects of a combined exercise intervention (aerobic + resistance exercise) in the aerobic fitness, muscle strength and muscle-tissue adaptations at the molecular level of the Harlequin (*Hq*) mutant mouse. In this animal, proviral insertion in the X-linked gene encoding Apoptosis Inducing Factor (AIF) produces a large decrement (~80%) in the expression of this mitochondrial protein (27), leading to respiratory chain complex I deficiency (28) and to a phenotype that mimics the clinical features of affected patients, including skeletal muscle myopathy (29–31).

## Materials and Methods

### Animals

All experimental protocols were approved by the institutional ethics committee (project number 111/15) and were conducted in accordance with European (European convention ETS 123) and Spanish (32/2007 and R.D. 1201/2005) laws on animal protection in research.

**Figure 1:**
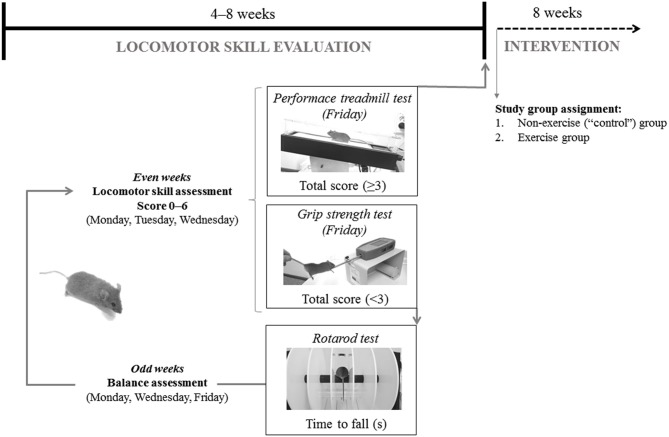
Representation of mice baseline evaluation before group assignment.

Heterozygous 6–8 week-old, male Harlequin mice (*n* = 21, *Hq*/Y named “*Hq*” hereafter, B6CBACa Aw-J/A-Aifm1Hq/J) and gender and age-matched wild-type (WT) mice (*n* = 12) of the same genetic background (B6CBACa Aw-J/A), were obtained from The Jackson Laboratory (Bar Harbor, ME). Animals were housed at 21°C and 60% humidity with a 12-h light/dark cycle and with free access to food and water in the animal facility of *Hospital Universitario 12 de Octubre* (Madrid, Spain). To ensure that all mice presented with the same degree of disease development at the moment of their incorporation into the study groups, each *Hq* mouse was previously evaluated from 8 weeks of age until the first signals of ataxia appeared following a locomotor skill and balance evaluation (Figure 1). The data obtained from the WT animals during the same period were used as a reference for absence of the aforementioned alterations as well as for biochemical analyses (see below).

Locomotion analysis was assessed while the mouse walked at a speed of 10 cm·s^−1^ (15 min, 15% inclination) on a treadmill (Harvard Apparatus; Panlab, Barcelona, Spain) during the even weeks of the locomotor skill evaluation phase. All mice were allowed to adapt to the treadmill in three familiarization sessions. An electric grid was placed at the end of the treadmill to deliver electrical shocks of constant intensity to encourage the animals to continue running on the treadmill. These sessions involved a gradual increase in running time and intensity, starting just placing the mice on the treadmill the first day: 0% inclination and 0 cm·s^−1^ speed for 1 min, with no electrical stimulation, and ending with a 15 min running period at low intensity on the third day (15% and 10 cm·s^−1^, electrical stimulation 0.1 mA, 1 Hz, 200 ms). Two examiners scored the motor function of each mouse and, if two scores did not match, the lowest score was chosen. Scores ranged from 0 (absence) to 2 (maximal severity grade) in the following manner: (i) walking gait balance, 0, 1, or 2 for smooth gait pattern, mild imbalanced and unstable walking gait, respectively; (ii) 0, 1, or 2 for ability to walk straight, first signs of diagonal walk and gait instability, or inability to walk straight, respectively; (iii) alterations in hind limb gait, 0, 1, or 2 for normal coordination, first signs of alterations in the stride frequency, and lack of hind left-right limb coordination, respectively. A total score (0–6) was generated by summing individual scores. Data from the 3 days was averaged and if the score was <3, the animals continued in the locomotor skill evaluation phase and performed the grip strength test (described below). Conversely, animals with an average score ≥3 were included in the training phase and were subjected to a treadmill performance test (described below).

During the locomotor skill evaluation phase, cerebellar ataxia was also tested, using the rotarod device (Letica Scientific Instruments; Panlab, Barcelona, Spain) on alternative days (odd weeks, 3 days in total) to avoid fatigue and following the protocol described elsewhere (27). To familiarize with the apparatus, mice underwent a training session of three trials (60 s each) in which the rod was kept stationary for the first trial and held at 4 rpm for the last two trials (32).

### Group Assignment

**Figure 2:**
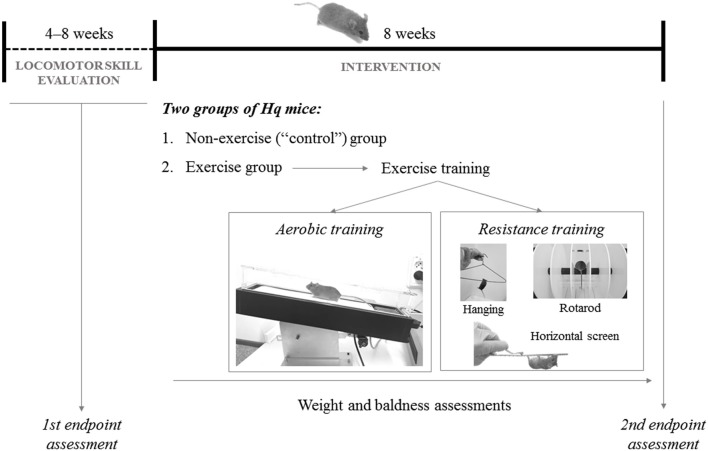
Representation of the study design.

*Hq* mice were paired-matched based on their aerobic fitness in the treadmill test (see below for a description of the assessment method) at baseline. Thus, among each pair of *Hq* mice with similar performance in the pre-intervention aerobic treadmill tests, one mouse was randomly assigned to an *exercise* group (*n* = 10), subjected to an 8 week exercise training program, and the other to a *non-exercise* (control) group (*n* = 11), allowed to freely move in the cage, but not performing the program (Figure 2). On the other hand, none of the WT mice used as reference underwent exercise training (i.e., same treatment as for the *Hq* control group).

### Exercise Training

The exercise intervention included 5 weekly sessions (Monday–Friday; session duration of 40–60 min), each including aerobic and resistance training.

#### Aerobic Training

*Hq* mice were allowed to adapt to the treadmill (Harvard Apparatus; Panlab, Barcelona, Spain) before their incorporation into the exercise intervention, as previously described (33). Exercise duration, treadmill speed, and inclination were gradually increased during the program following a defined interval training protocol from our group, with slight modifications (33). Briefly, beginning at very low workloads in the first session (20 min at 35% of the maximal velocity obtained during the aerobic fitness test that is described below) and 0% gradient on the first day, and ending with 40 min at 65–70% of maximal velocity and 15% gradient in the last sessions. Only gentle tail touching was used to prompt the mice to run, and no electrical stimulation was applied during the training sessions.

#### Resistance Training

This followed the aerobic training and included three exercises: (i) horizontal screen exercise (Monday); (ii) hanging exercise with two limbs (Wednesday and Friday); and (iii) rotarod exercise (Tuesday and Thursday). For (i), the *Hq* mouse was placed inverted on top of a screen and had to climb back over to the top. When this was achieved, the mouse was placed again in the initial position. The number of repetitions was gradually increased (from 1 to 6), each also of increasing duration (from 30 s at the beginning to 90 s at the end of the training phase), with a constant 2 min rest period between repetitions. For (ii), *Hq* mice were picked up by the tail and placed on a metal cloth hanger taped to a shelf and maintained at 40 cm above a layer of bedding to cushion the falls (32). Mice were allowed to grasp the wire only using the two forepaws for as long as they could during one set of six repetitions, with a 10 min rest period between them. For (iii), each *Hq* mouse was placed onto the rod in a rotarod device (Letica Scientific Instruments, Panlab; Barcelona, Spain) and walked the rotating rod at speeds that increased from 4 to 40 rpm over a 300 s period. When the first fall occurred, the number of rpm was recorded, and the mouse exercised at this speed during 5 min (32).

### Measurements

Study endpoints were assessed at the start and the end of the exercise intervention except for muscle molecular measurements, which could only be measured at the end. Serum samples were obtained before and after concluding the exercise program. Forty eight hours after the last exercise test, *Hq* mice were killed by cervical dislocation and the *biceps femoris* and *quadriceps femoris* muscles were dissected and immediately snap-frozen in liquid nitrogen before storage at −80°C until molecular analysis. The WT mice were sacrificed with the aforementioned procedure at the same time point.

#### Aerobic Fitness

An incremental treadmill test was used to determine the maximal aerobic fitness (expressed as the total distance [meters] run by the mice) of *Hq* mice (33). The test was performed after a warm-up period of 10 min at a speed of 10 cm·s^−1^ (with 15% inclination) and followed a previous protocol from our group with slight modifications in workload increases (33). Thus, the initial velocity was 5 cm·s^−1^ and was increased in 2 cm·s^−1^ every 2 min until exhaustion, with a constant treadmill inclination of 15% during the whole test. Mice were considered exhausted when they spent more than 5 continuous s on the electric grid and were unable to continue running at the next speed (33).

#### Forelimb Grip Strength

Grip strength of *Hq* mice was measured using an isometric force transducer (Harvard apparatus; Panlab, Barcelona, Spain). Maximum force (grams) exerted by the mouse before losing grip was recorded in three trials (each separated from the next by a 5 min interval). The best score for each animal was recorded as the maximal grip strength.

#### *Hq* Phenotype

Cerebellar ataxia in *Hq* mice was tested on the rotarod device. Each mouse walked the rotating rod at speeds that increased from 4 to 40 rpm over a 300 s period (27). The latency to fall from the rotating rod was recorded in each trial. The best of the 3 day latency was considered for analysis. We additionally evaluated two other hallmarks of the *Hq* phenotype (27) weekly throughout the study: (i) hair loss, which was assessed as the percentage of body surface area without hair (30), and (ii) weight.

#### Serum Determinations

Muscle damage in *Hq* mice was assessed through the measurement of creatine kinase (CK) activity in serum samples (Creatine Kinase Activity Assay Kit; Sigma Aldrich, MO).

#### Tissue Processing

Muscle tissue of both *Hq* and WT mice was either processed to obtain total homogenates as described previously (33), or processed to obtain mitochondria-enriched fractions with a specific kit (Mitochondria Isolation Kit for Tissue; Abcam, UK) according to manufacturers' instructions.

#### Western Blotting

Aliquots of total homogenates or mitochondria-enriched fractions containing equal amount of protein were resolved by sodium dodecyl sulfate polyacrylamide gel (SDS-PAGE) electrophoresis, transferred to polyvinylidene difluoride (PVDF) membranes, blocked, and incubated with primary antibodies for the determination of levels of the following proteins: catalase (CAT), citrate synthase (CS), cytosolic superoxide dismutase (cSOD), gluthathione reductase (GR), mitochondrial superoxide dismutase (mtSOD), mitochondrial transcription factor A (TFAM), NADH-ubiquinone oxidoreductase subunit B8 (NDUFB8) and S1 (NDUFS1), peroxisome proliferator-activated receptor-gamma co-activator 1α (PGC-1α), and ribosomal protein S6 kinase beta-1 total (P70S6K) or phosphorylated at threonine 389 (P70S6K Thr389, or simply “phosphorylated” pP70S6K) (Supplementary Table 1). After incubation with peroxidase-conjugated secondary antibodies, immunoreactive bands were detected with the ECL Prime Western Blotting Detection Reagent (Amersham Biosciences, UK) in a ChemiDoc™ MP Imager (Bio-Rad, Hercules, CA). Band densities were evaluated by densitometric scanning [ImageJ software, National Institutes of Health (34)]. Either glyceraldehyde-3-phosphate dehydrogenase (GAPDH) was immunodetected and used as loading control for total homogenates, or total protein load per lane was determined with Coomassie Blue staining of the PVDF membranes (35). When needed, membrane stripping was performed before immunodetection of GAPDH.

#### Spectrophotometry

Activities of OXPHOS complexes were determined in muscle total homogenates prepared in 225 mM mannitol, 75 mM sucrose, 0.1 mM EDTA, and 10 mM Tris–HCl pH 7.4, according to standardized protocols for spectrophotometric assays (36), and in mitochondria-enriched fractions by a researcher blinded to the mouse group (control *Hq*, exercised *Hq*, or WT) from which the samples were obtained. For complexes II–IV, 20 μg of protein was used in total homogenates, and 6 μg in mitochondria-enriched fractions, whereas 60 μg of protein was added to the assay for complex I in total homogenates, and 10 μg in mitochondria-enriched fractions. Complex V activity was assayed as F1-ATPase using 20 μg of protein according to a previously described method (37). CS activity, was spectrophotometrically determined at 30°C in the presence of 0.1% Triton X-100 following the formation of 5-thio-2-nitrobenzoic acid at 412 nm, as previously described (38), using 60 μg of protein in total homogenates and 6 μg in mitochondria-enriched fractions.

All the aforementioned muscle biochemical variables obtained with western blotting or spectrophotometry in samples of *Hq* mice were expressed relative to the corresponding mean values for the WT mice.

#### Histochemistry

For muscle histology *tibialis anterior* muscles were frozen in N_2_-cooled isopentane, and hematoxylin-eosin staining on transverse sections of muscle was performed on cryostat sections according to standard techniques (39), and cross-sectional area of fibers was determined with Image J software.

#### Statistical Approach

Data are presented as mean ± standard error of measurement (SEM). The normal distribution (Shapiro-Wilk test) and homoscedasticity (Levene's test) of the data were checked before any statistical treatment. A two-factor (group [exercise, control] by time) ANOVA with repeated measures on time was used for between-group comparisons of outcomes with more than one measurement over time (i.e., aerobic fitness, muscle strength, balance, serum CK, hair loss and weight). To minimize the risk of type I error, *post hoc* comparisons were only performed within-groups (with the Bonferroni test) when a significant interaction group by time was found. The non-parametric Mann–Whitney's *U*-test was used for between-group comparisons of outcomes assessed at one single time point (i.e., muscle biochemical analyses). All statistical analyses were performed with the SPSS 23.0 package (SPSS, Inc., Chicago, IL) setting the significance level at 0.05.

## Results

### Endpoints With Repeated Measurements in Time

A significant group by time interaction effect was found for aerobic fitness (*p* = 0.014, effect size [η^2^] = 0.304, power = 0.728), with *post hoc* analyses indicating an improvement after the training intervention (*p* = 0.016) and no change in control mice after the same time period (*p* = 0.277) (Table 1). A significant group by time interaction effect was also observed for muscle strength (*p* = 0.037, η^2^ = 0.209, power = 0.566), which increased in the exercise group (*p* = 0.003) but did not change in control mice (*p* = 0.751). Individual data on aerobic fitness and muscle strength by group are shown in Supplementary Figure 1. On the other hand, no significant group by time interaction was found for time to fall (*p* = 0.544), serum CK (*p* = 0.290) (Table 1), body weight (*p* = 0.058), or hair loss (*p* = 0.406) (Supplementary Figure 2).

**Table 1:** Effects of the exercise intervention on study endpoints with repeated measurements before and after the intervention.

**Endpoint**	**Group**	***N* with data**	**Pre-training**	**Post-training**	**Group (*p*)**	**Time (*p*)**	**Group by time interaction (*p*)**
Aerobic fitness (distance, in m)	Control Exercise	10 9	191 ± 40 176 ± 18	158 ± 37 259 ± 43^*^	0.372	0.256	**0.014**
Strength (forelimb test, g)	Control Exercise	11 10	109 ± 9 110 ± 10	111 ± 8 126 ± 10^†^	0.537	**0.015**	**0.037**
Balance (time to fall, in s)	Control Exercise	11 10	59 ± 12 79 ± 14	23 ± 7 52 ± 6	0.073	** <0.001**	0.544
Serum CK (U/L)	Control Exercise	11 9	93 ± 14 94 ± 33	96 ± 8 106 ± 21	0.976	0.499	0.290

*Data are mean ± standard error of the mean. Significant p-values (<0.05) are highlighted in bold. CK, creatinekinase*.

* p = 0.016 for pre vs. post;

† *p = 0.003 for pre vs.post*.

### Endpoints With Single Measurement in Time

Higher levels of total P70S6K and pP70S6K were observed at the end of the training intervention in the exercise group compared with the control group (Table 2; Supplementary Figure 3A), which also showed high levels compared to the wild-type animals, as well as a non-significant trend toward a higher pP70S6K/ P70S6K ratio (Table 2; Supplementary Figure 3A). Exercise resulted in higher levels of PGC-1α and a tendency toward higher levels of TFAM (Table 2; Supplementary Figure 3B).

**Table 2:** Biochemical variables in *Hq* mice.

	**Variable**	***N* with data (control/exercise)**	**Control group**	**Exercise group**	***P*-value Mann-Whitney's *U*-test**
Anabolism protein levels in *Biceps femoris* homogenates (% of WT)	P70S6K	10/9	168 ± 20	292 ± 16	** < 0.001**
	pP70S6K	10/9	156 ± 30	249 ± 30	**0.028**
	pP70S6K/P70S6K	10/9	91 ± 3	109 ± 6	0.095
Mt biogenesis protein levels in *Biceps femoris* homogenates (% of WT)	PGC-1α	5/4	82 ± 7	126 ± 19	**0.032**
	TFAM	11/10	83 ± 3	94 ± 4	0.060
OXPHOS activities in *Biceps femori*s homogenates (% of WT)	Complex I	10/9	75 ± 4	95 ± 6	**0.019**
	Complex II	10/9	82 ± 5	94 ± 5	0.161
	Complex III	1109	79 ± 5	97 ± 4	**0.031**
	Complex IV	10/9	88 ± 4	102 ± 4	0.136
	Complex V	10/9	77 ± 9	105 ± 9	**0.024**
	Citrate synthase	10/8	79 ± 3	91 ± 4	0.059
OXPHOS protein levels in *Biceps femoris* homogenates (% of WT)	ATP5a	11/9	86 ± 12	98 ± 13	0.387
	UQCRC2	11/9	77 ± 9	91 ± 21	0.705
	MTCO1	11/9	76 ± 13	108 ± 25	0.529
	SDHB	11/9	86 ± 5	85 ± 4	0.468
	NDUFB8	11/9	77 ± 6	81 ± 10	0.809
	NDUFS1	10/8	85 ± 5	93 ± 5	0.095
	Citrate synthase	11/9	84 ± 4	124 ± 14	0.132
OXPHOS activities in *Quadriceps* mitochondrial fraction (% of WT)	Complex I	10/5	87 ± 12	93 ± 19	0.953
	Complex II	5/5	89 ± 12	70 ± 7	0.548
	Complex III	5/5	117 ± 16	86 ± 14	0.310
	Complex IV	4/5	104 ± 7	99 ± 8	0.811
	Citrate synthase	5/5	118 ± 10	117 ± 17	0.905
OXPHOS protein levels in *Quadriceps* mitochondrial fraction (% of WT)	ATP5a	4/5	108 ± 14	125 ± 20	0.343
	UQCRC2	4/5	123 ± 12	146 ± 14	0.486
	MTCO1	4/5	118 ± 4	119 ± 8	0.886
	SDHB	4/5	99 ± 2	94 ± 10	1.00
	NDUFB8	4/5	79 ± 9	74 ± 10	0.886
	NDUFS1	4/5	30 ± 3	21 ± 6	0.343
Antioxidant enzymes protein levels in *Biceps femoris* homogenates (% of WT)	mtSOD	11/10	94 ± 13	94 ± 5	0.607
	cSOD	11/10	120 ± 20	97 ± 10	0.557
	GR	11/10	99 ± 4	109 ± 7	0.512
	CAT	11/10	204 ± 22	141 ± 23	**0.036**

*Data (mean ± SEM) are expressed and analyzed as a percentage of mean value of an age- and gender-matched matched non-exercised wild type (WT) group (n = 4–12). Significant p-values are highlighted in bold. ATP5A, ATP synthase alpha subunit; CAT, catalase; cSOD, cytosolic superoxide dismutase; GR, glutathione reductase; Mt, mitochondrial; MTCO1, mitochondrial cytochrome c oxidase subunit I; mtSOD, mitochondrial superoxide dismutase; NDUFB8, ubiquinone oxidoreductase subunit B8; NDUFS1, ubiquinone oxidoreductase subunit S1; P70S6K, total ribosomal protein S6 kinase beta-1; pP70S6K, ribosomal protein S6 kinase beta-1 phosphorylated at threonine 389; PGC-1α, proliferator activated receptor gamma co-activator 1α; SDHB, succinate dehydrogenase complex iron sulfur subunit B; TFAM, transcription factor A mitochondrial; UQCRC2, ubiquinol-cytochrome C reductase core protein 2*.

The activities of the OXPHOS complexes I, III, and V measured in muscle homogenates were higher in the exercised than in the control group and a trend toward significant differences was also found for CS activity (Table 2). However, no significant between-group differences were found for the muscle protein levels of representative subunits of the different OXPHOS complexes or for CS as measured by western blotting in total homogenates (Table 2; Supplementary Figure 3C), the enzymatic activity of respiratory chain complexes, or the representative OXPHOS proteins measured in mitochondria-enriched fractions (Table 2; Supplementary Figure 3D).

Exercised mice presented with lower CAT levels at the end of the training intervention compared to control mice, which showed abnormally high levels compared to the wild-type mice with no between-group differences for the remainder of antioxidant enzymes (Table 2; Supplementary Figure 3E).

To further assess the time course of the myopathy in the *Hq* mice, we analyzed forelimb strength in an independent cohort of *Hq* and WT mice from 1.5 to 3 months of age, with the results showing significantly lower muscle strength in the former since 1.5 months (Supplementary Figure 4). Finally, to study the effects of exercise training and P70S6K activation on skeletal muscle mass, we studied the cross-sectional fiber area of *tibialis anterior* muscles from some WT and *Hq* mice. The results indicated lower cross-sectional area in both control and exercise *Hq* animals compared to WT (Supplementary Figure 5).

## Discussion

The present study shows that an exercise intervention combining aerobic and resistance training induced significant benefits on the aerobic fitness and muscle strength of *Hq* mice, partially counteracting the myopathy progression that characterizes the time-course of the disease. These improvements were accompanied by mild increases in the activity of OXPHOS complexes I, III, and V, and by a trend to higher CS activity in total homogenates. Further, the intervention did not induce major skeletal muscle damage, as reflected by the lack of differences between groups in a well-accepted marker of this phenomenon, serum CK activity. No benefits were noted on phenotypes not directly related to muscle tissue such as cerebellar ataxia (i.e., balance performance) or growth impairment, which, as opposed to the aforementioned fitness/muscle variables, showed a similar evolution over time in the two groups. Of note, the fact that the exercise training program was initiated when the first signs of ataxia and myopathy were already present might explain, at least partly, why we did not find greater improvements in muscle biomarkers. In this respect, however, a strength of our design is that it mimics a frequent situation in patients, that is, the treatment starts when the disease phenotype is already well-established.

The *Hq* mouse model replicates the main features of respiratory chain complex I deficiency in humans (30), which is the most common respiratory chain defect (40). *Hq* mutant mice not only mimic clinical features of this condition in important aspects such as tissue specificity, disease onset and course, or inter-individual variability (30), but also show disturbances frequently found in other diseases associated with respiratory chain deficiencies such as ataxia, myopathy and retinal degeneration (30). Therefore, this model has been considered a valuable tool for assessing potential therapeutic approaches in MD, and previously nutritional, genetic, and pharmacological interventions, have been proven effective to attenuate several disease-related markers (41–44). In this regard, muscular weakness, the main feature of myopathy, is an early finding in the *Hq* mouse model, in which we observed weakness since 1.5 month of age. In turn, the latter result correlates well with the finding of complex I deficiency in skeletal muscle previously reported in 1 month-old *Hq* mice, a molecular phenotype trait that remains during the course of the disease (30). The early appearance of myopathy in *Hq* mice also makes this model a valuable tool for the study of exercise training as therapy for MD. Our results showing physical capacity improvements in trained *Hq* are in agreement with previous studies analyzing exercise training as therapy in other mouse models of MD. For example, Wenz et al., reported a lower number of falls during a treadmill test after a 1.5 month training program in a mouse model of cytochrome oxidase deficiency (23). After a 1 month training program, “Mutator” mice were able to reach levels of treadmill running performance similar to those of healthy (WT) animals (25). Our results demonstrating improvements in the physical capacity of *Hq* mice in response to exercise training are novel for this strain, and might allow to gain insight into the molecular underpinnings of exercise-induced adaptations in this model.

The promotion of mitochondrial biogenesis through either genetically or pharmacologically (bezafibrate administration) stimulation of PGC-1α, has been shown to increase OXPHOS capacity in *cytochrome c oxidase-*deficient mice (9). Accordingly, exercise training-induced promotion of mitochondrial biogenesis through the activation of PGC-1α, has also proven to mediate the enhancement of oxidative capacity in animal (23, 25) and patient studies of MD (11–13). In this regard, in the present study, exercise training slightly improved muscle levels of PGC-1α and TFAM, and complex I, III, and V enzyme activities in muscle homogenates of *Hq* mice. Additionally, CS activity, which is a common marker of mitochondrial content, also showed a trend to increase in this group. Overall, these results indicate that exercise training was able to slightly promote mitochondrial biogenesis in the *Hq* mice, leading to partial normalization of the altered oxidative capacity. Moreover, we observed higher activities of OXPHOS complexes in muscle tissue homogenates but not in mitochondria-enriched fractions, reflecting that the results in the homogenates of the exercised *Hq* were due to a higher content of mitochondria rather than to a higher density of OXPHOS complexes per mitochondria. Therefore, our results support the role of mitochondrial biogenesis as a mediator of the beneficial effects of physical exercise on muscle OXPHOS capacity in the *Hq* mice. Of note, administration of the PGC-1α agonist bezafibrate has failed to improve mitochondrial biogenesis or muscle strength in *Hq* mice, with this treatment in fact inducing liver disease as a major adverse effect (41). Thus, up to date exercise training appears as the safest and most effective strategy to induce mitochondrial biogenesis in the *Hq* mouse model.

One aspect of our study that must be highlighted is the novelty of the exercise intervention, which included both endurance and resistance training. Previous studies in animals and patients with MD have focused on aerobic exercise owing to its potential to promote mitochondrial biogenesis and eventually increase aerobic fitness (11–13, 16–18, 45). Resistance training, however, might not only improve OXPHOS capacity (19) but also provide benefits on muscle mass and strength (20), and indeed we recently observed improvements with this type of intervention not only in the aerobic fitness, but also in the muscle strength and mass of patients with MD (14). Here, we also observed training-induced increases in muscle OXPHOS capacity and a trend to higher CS activity [being a classical marker of muscle aerobic adaptation and upregulated mitochondrial biogenesis (46)], and also in muscle strength. The higher levels of activation of P70S6K (i.e., p70S6K) and the trend toward higher levels of the ratio p70S6K/total P70S6K (a marker of muscle anabolism) observed in the exercise group could also support the role of combined resistance and aerobic exercise in the promotion of muscle anabolism, as P70S6K (a downstream effector of the mammalian target of rapamycin [commonly known as “mTOR”] pathway) is involved in protein synthesis and consequently muscle mass growth (47). Of note, the benefits observed in muscle strength are of particular relevance given that the *Hq* phenotype is characterized by marked muscle atrophy (i.e., lower muscle cross-sectional area and strength, and lower number of fast-twitch fibers compared to wild-type mice) (29, 48), and we recently showed that patients with MD also present with a lower (−18%) lean mass than their healthy peers (14). Nevertheless, preliminary assessment of fiber cross-sectional area in the *tibialis anterior* of control and exercised animals suggested no differences between both groups and thus no major occurrence of exercise-promoted skeletal muscle anabolism. In this respect, studies involving patients with MD have also demonstrated improvements in physical performance and strength without increases in the cross-sectional area of muscle fibers in response to training (21).

Another interesting finding of our study were the lower levels of the antioxidant enzyme CAT found in the exercised mice. *Hq* mice typically present with an 80% decrease in AIF, a protein that might be involved not only in apoptosis and OXPHOS complexes I and IV biogenesis (49, 50), but also in facilitating cellular redox homeostasis (27). Specifically, AIF has been proposed to exert an antioxidant activity as a peroxide scavenger, with reduced AIF levels resulting in increased peroxide sensitivity and consequently in greater levels of oxidative damage and cell death (27). Elevated levels of oxidative stress—as reflected by increased CAT activity in the cerebellum, heart, and skeletal muscle—have been previously reported in *Hq* mice and in other AIF-deficient mouse models (27, 51), which is in agrement with our results (i.e., the CAT levels of control *Hq* mice were significantly higher [by ~2-fold] than those of wild-type mice). Our results, therefore, suggest that the exercise training intervention might have contributed to improve redox status, with the antioxidant levels of exercised *Hq* mice resembling more those of wild-type mice compared to their non-exercise pairs.

The present study has some limitations. First, randomization, allocation concealment, and blinding outcome assessment can reduce the risk of bias in animal studies. In this respect, although we could not adhere to these recommendations, we followed some measures to limit bias. First, we pair-matched mice in both groups based on their baseline aerobic fitness. Second, researchers in charge of muscle biochemical assessments were blinded to the mouse group from which samples were taken. Furthermore, we limited the risk of statistical error type I by using non-parametric statistical tests unpaired comparisons between exercise and control group, respectively. Another limitation is that we did not assess the effects of exercise training on muscle mass, which would have provided additional information, and we only performed preliminary assessment of muscle fiber cross-sectional area in a few mice. In turn, one of the major novelties of our study was the type of intervention applied, as to our knowledge this is the first study in which a mouse model of MD is subjected to resistance training. Moreover, we assessed both functional (i.e., fitness status) and biological (OXPHOS activity) markers and analyzed potential molecular pathways involved in these adaptations, which we consider as a major strength of our study.

## Conclusions

In summary, an intervention combining aerobic and resistance exercises improved markers of physical capacity (aerobic fitness and muscle strength) in *Hq* mutant mice, a model of respiratory chain complex I deficiency. In turn, this intervention induced mild increases in the activation of signaling pathways involved in mitochondrial biogenesis (i.e., PGC-1α) and muscle anabolism (i.e., pP70S6K), muscle OXPHOS activity, and redox status–as reflected by a decrease in CAT levels. Although more research is needed, these results are encouraging and might provide useful information for the management of MD.

## Data Availability

Datasets are available on request to the authors.

## Ethics Statement

All experimental protocols were approved by the institutional ethics committee (project number 111/15) and were conducted in accordance with European (European convention ETS 123) and Spanish (32/2007 and R.D. 1201/2005) laws on animal protection in research.

## Author Contributions

CF-L: study design, experimental procedures, and drafting of the manuscript. PV: data analysis and drafting of the manuscript. SL-M, MF-T, VB-G, and LR-V: experimental procedures. JA, MAM, and AL: data analysis and manuscript edition. MM: study design, drafting of the manuscript, and direction. All authors revised the manuscript critically for important intellectual content and approved the final version.

### Conflict of Interest Statement

The authors declare that the research was conducted in the absence of any commercial or financial relationships that could be construed as a potential conflict of interest.
