# Supplementary material for: Physical Exercise and Mitochondrial Disease: Insights From a Mouse Model
Source: Front Neurol. 2019 Jul 25;10:790. doi: 10.3389/fneur.2019.00790 (PMC6673140; doi:10.3389/fneur.2019.00790)
Supplement: Supplementary file 1 [file Table_1.DOCX]

**Supplementary Table 1**. Primary antibodies used in this study.

| **Antibody against** | **Provider** | **Reference** | **Link** |
| --- | --- | --- | --- |
| CAT | Sigma | C0979 | https://www.sigmaaldrich.com/catalog/product/sigma/c0979?lang=es&region=ES |
| CS | Abcam | ab96600 | http://www.abcam.com/citrate-synthetase-antibody-ab96600.html |
| cSOD | Enzo | ADI-SOD-100 | http://www.enzolifesciences.com/ADI-SOD-100/cu-zn-sod-polyclonal-antibody/ |
| GAPDH | Sigma | G9545 | https://www.antibodypedia.com/gene/3923/GAPDH/antibody/80237/G9545 |
| GR | Abcam | ab16801 | https://www.abcam.com/glutathione-reductase-antibody-ab16801.html |
| mtSOD | MERCKMillipore | 06-984 | http://www.merckmillipore.com/ES/es/product/Anti-Mn-SOD-Antibody,MM_NF-06-984?ReferrerURL=https%3A%2F%2Fwww.google.com%2F |
| NDUFB8 | Abcam | ab110242 | http://www.abcam.com/ndufb8-antibody-20e9dh10c12-ab110242.html |
| NDUFS1 | GeneTex | GTX113787 | https://www.antibodypedia.com/gene/34175/NDUFS1/antibody/177385/GTX113787 |
| PGC-1α | Cayman Chemicals | 101707 | Not available |
| TFAM | Abcam | ab131607 | https://www.abcam.com/tfam-antibody-ab131607.html |
| Total OXPHOS Rodent WB Antibody Cocktail | Abcam | ab110423 | http://www.abcam.com/total-oxphos-rodent-wb-antibody-cocktail-ab110413.html |
| P70S6K | Cell Signaling | #9202 | https://www.cellsignal.com/products/primary-antibodies/p70-s6-kinase-antibody/9202 |
| pP70S6K | Cell Signaling | #9205 | https://www.cellsignal.com/products/primary-antibodies/p70-s6-kinase-antibody/9202 |

Abbreviations: CAT, catalase; CS, citrate synthase; cSOD, cytosolic superoxide dismutase; GAPDH, glyceraldehyde-3-phosphate; GR, gluthathione reductase; mtSOD, mitochondrial superoxide dismutase; NDUFB8, NADH-biquinone oxidoreductase subunit B8; NDUFS1, NADH-ubiquinone oxidoreductase subunit S1; OXPHOS, oxidative phosphorylation system; PGC-1α, proliferator activated receptor gamma coactivator 1α; P70S6K, ribosomal protein S6 kinase beta-1; pP70S6K, ribosomal protein S6 kinase beta-1 phosphorylated at threonine 389; TFAM, transcription factor A mitochondrial.
