# Supplementary material for: Physical Exercise and Mitochondrial Disease: Insights From a Mouse Model
Source: Front Neurol. 2019 Jul 25;10:790. doi: 10.3389/fneur.2019.00790 (PMC6673140; doi:10.3389/fneur.2019.00790)
Supplement: Supplementary file 2 [file Presentation_1.PPTX]

## Slide 1
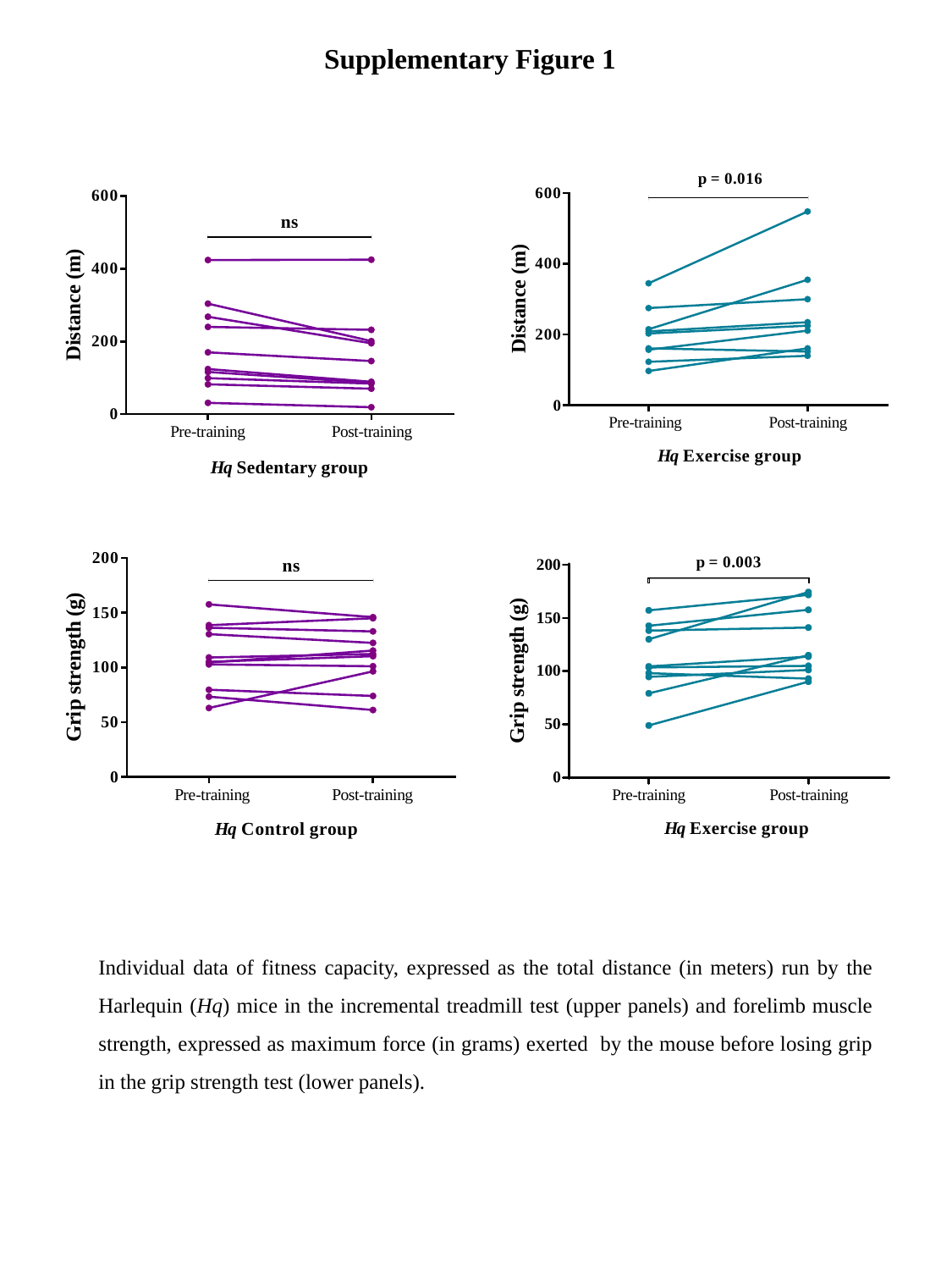

Supplementary Figure 1
Individual data of fitness capacity, expressed as the total distance (in meters) run by the Harlequin (Hq) mice in the incremental treadmill test (upper panels) and forelimb muscle strength, expressed as maximum force (in grams) exerted by the mouse before losing grip in the grip strength test (lower panels).

## Slide 2
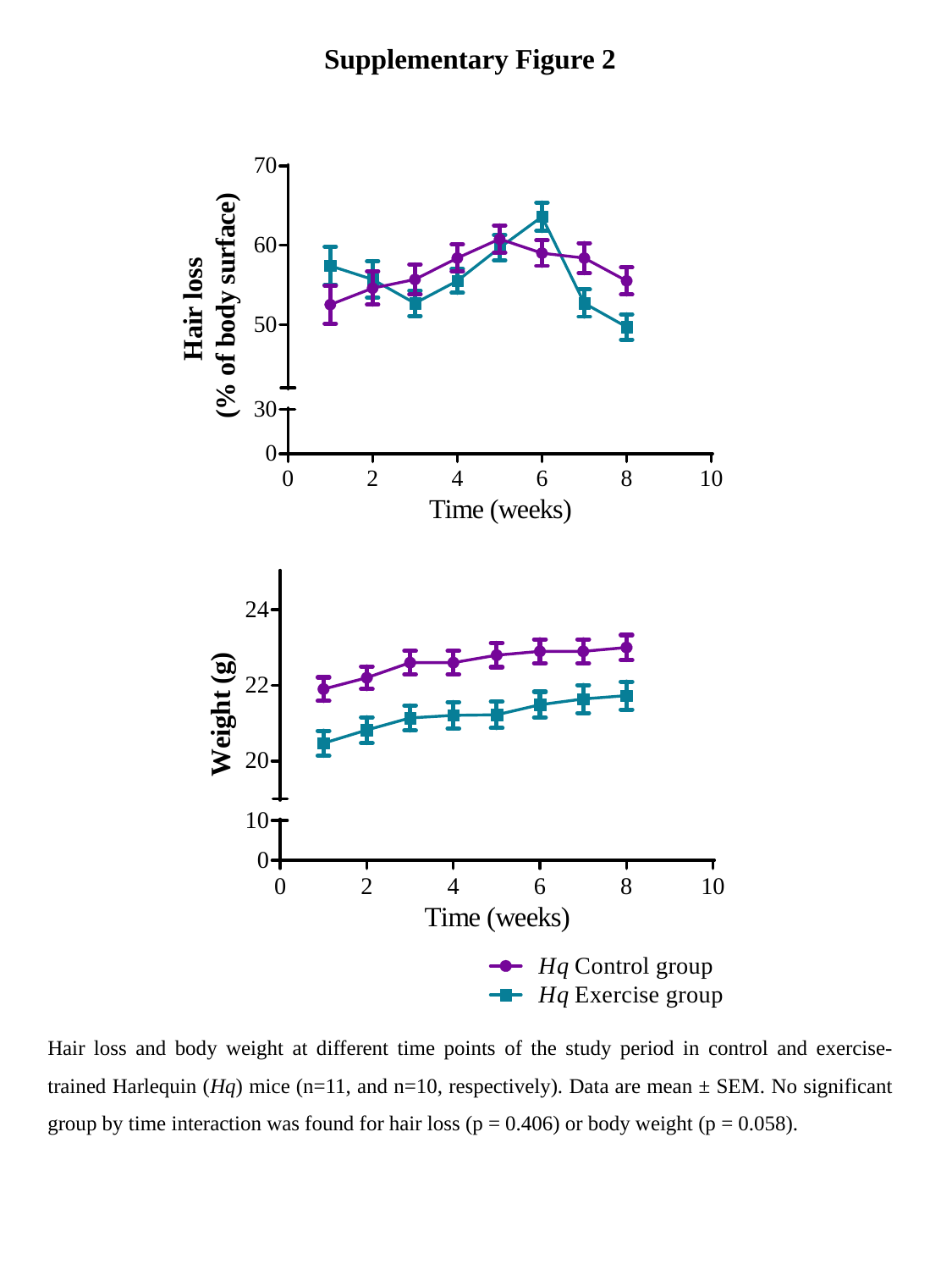

Supplementary Figure 2
Hair loss and body weight at different time points of the study period in control and exercise-trained Harlequin (Hq) mice (n=11, and n=10, respectively). Data are mean ± SEM. No significant group by time interaction was found for hair loss (p = 0.406) or body weight (p = 0.058).

## Slide 3
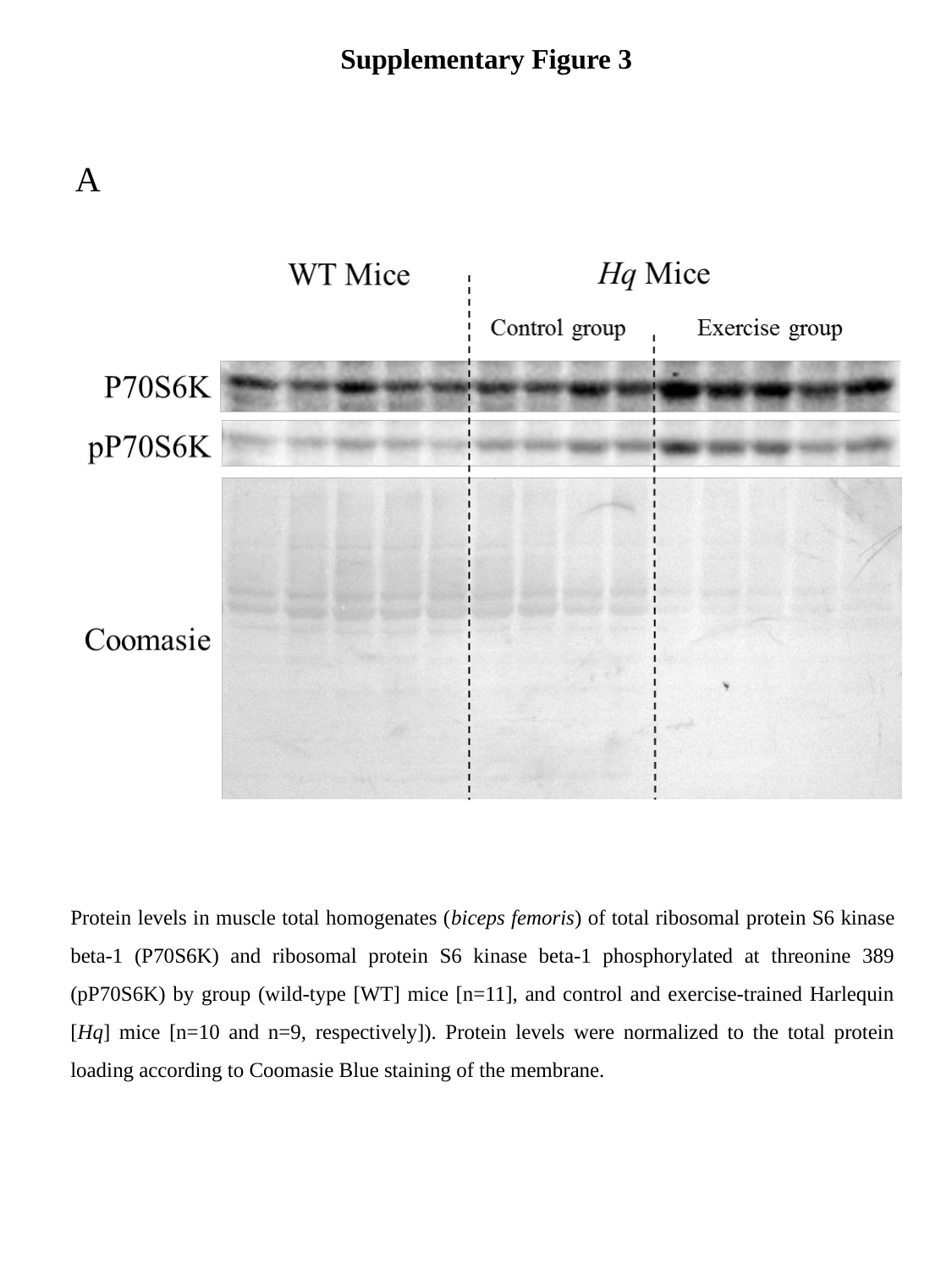

Supplementary Figure 3
A
Protein levels in muscle total homogenates (biceps femoris) of total ribosomal protein S6 kinase beta-1 (P70S6K) and ribosomal protein S6 kinase beta-1 phosphorylated at threonine 389 (pP70S6K) by group (wild-type [WT] mice [n=11], and control and exercise-trained Harlequin [Hq] mice [n=10 and n=9, respectively]). Protein levels were normalized to the total protein loading according to Coomasie Blue staining of the membrane.

## Slide 4
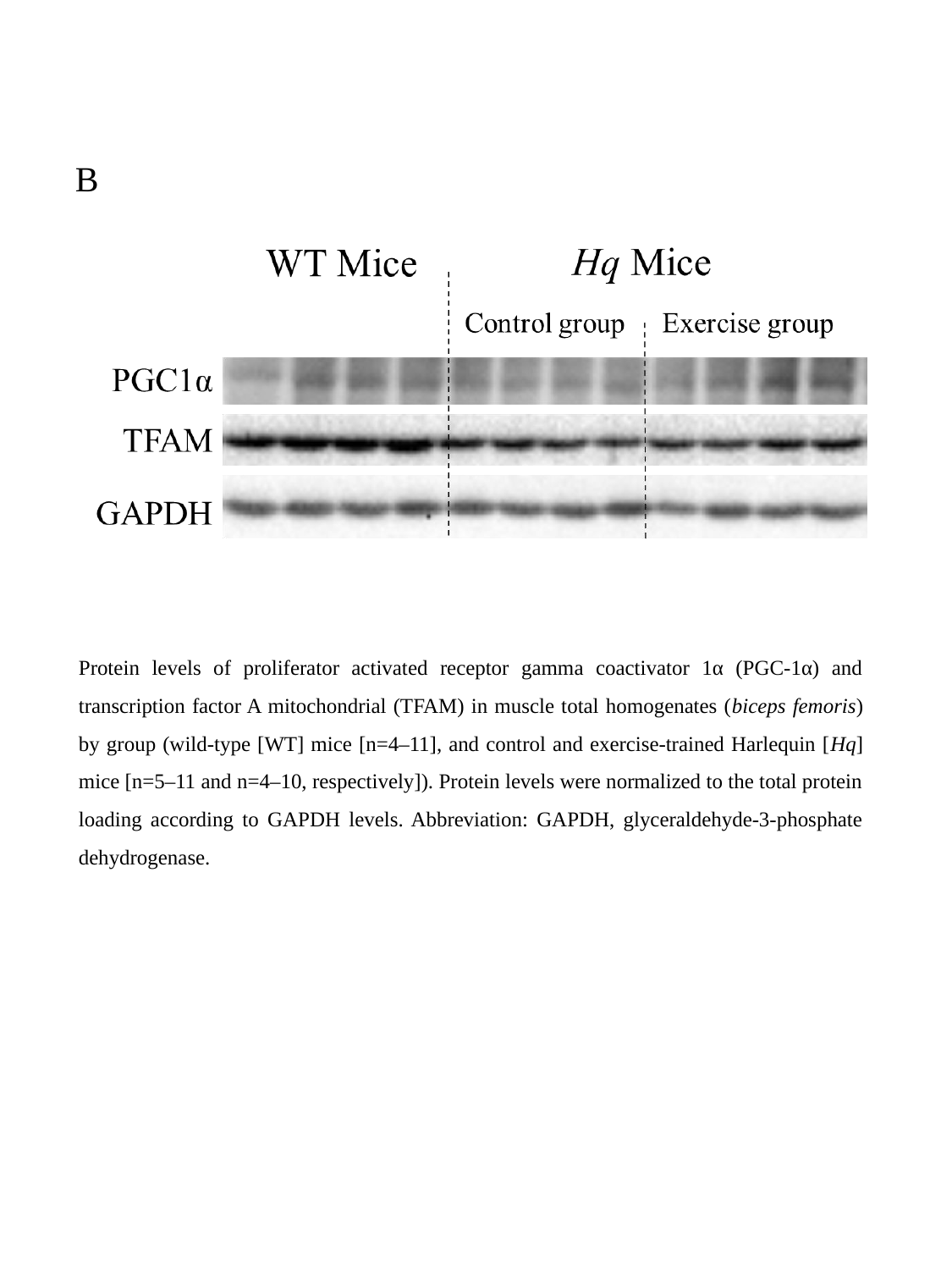

B
Protein levels of proliferator activated receptor gamma coactivator 1α (PGC-1α) and transcription factor A mitochondrial (TFAM) in muscle total homogenates (biceps femoris) by group (wild-type [WT] mice [n=4–11], and control and exercise-trained Harlequin [Hq] mice [n=5–11 and n=4–10, respectively]). Protein levels were normalized to the total protein loading according to GAPDH levels. Abbreviation: GAPDH, glyceraldehyde-3-phosphate dehydrogenase.

## Slide 5
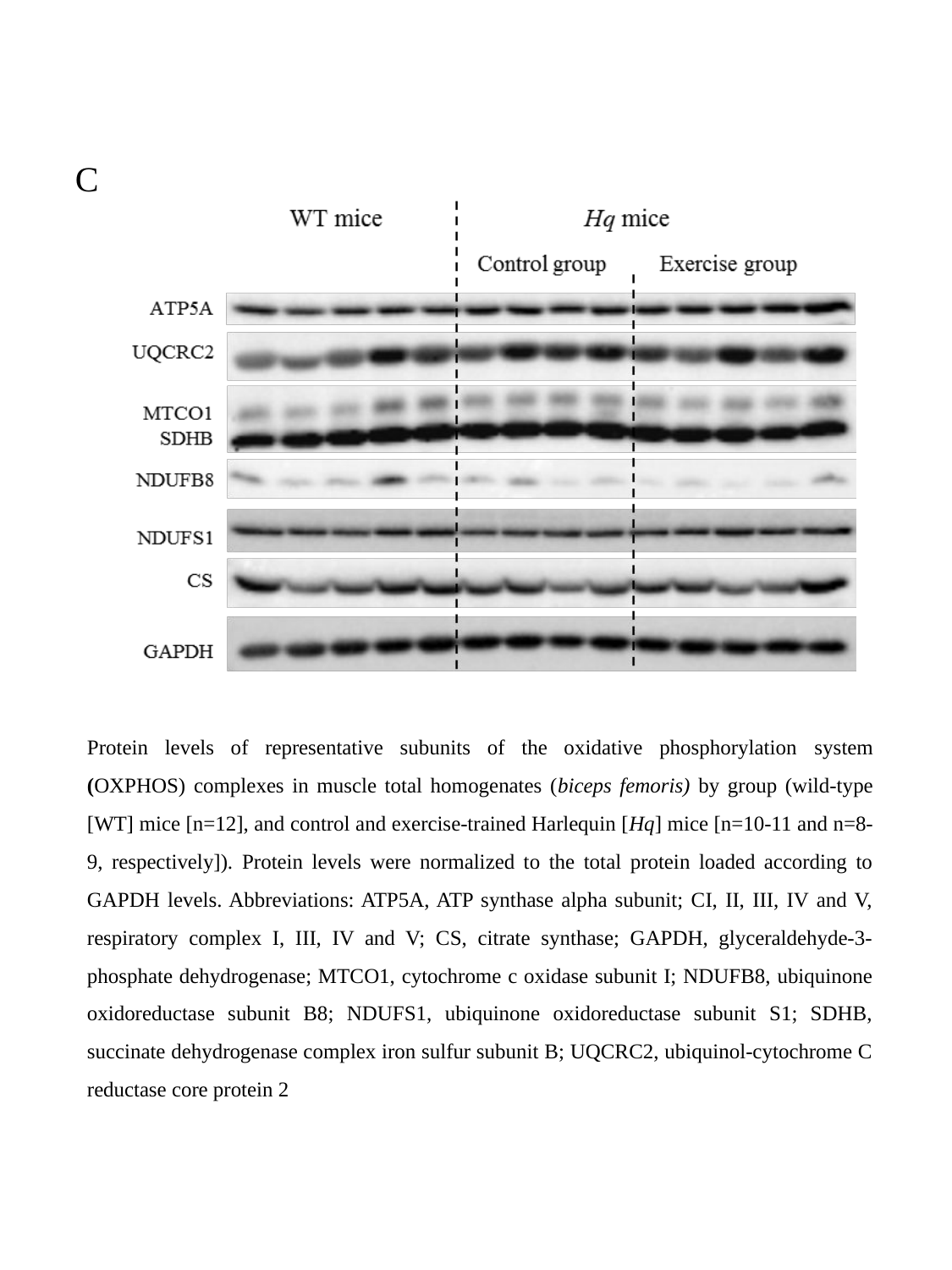

C
Protein levels of representative subunits of the oxidative phosphorylation system (OXPHOS) complexes in muscle total homogenates (biceps femoris) by group (wild-type [WT] mice [n=12], and control and exercise-trained Harlequin [Hq] mice [n=10-11 and n=8-9, respectively]). Protein levels were normalized to the total protein loaded according to GAPDH levels. Abbreviations: ATP5A, ATP synthase alpha subunit; CI, II, III, IV and V, respiratory complex I, III, IV and V; CS, citrate synthase; GAPDH, glyceraldehyde-3-phosphate dehydrogenase; MTCO1, cytochrome c oxidase subunit I; NDUFB8, ubiquinone oxidoreductase subunit B8; NDUFS1, ubiquinone oxidoreductase subunit S1; SDHB, succinate dehydrogenase complex iron sulfur subunit B; UQCRC2, ubiquinol-cytochrome C reductase core protein 2

## Slide 6
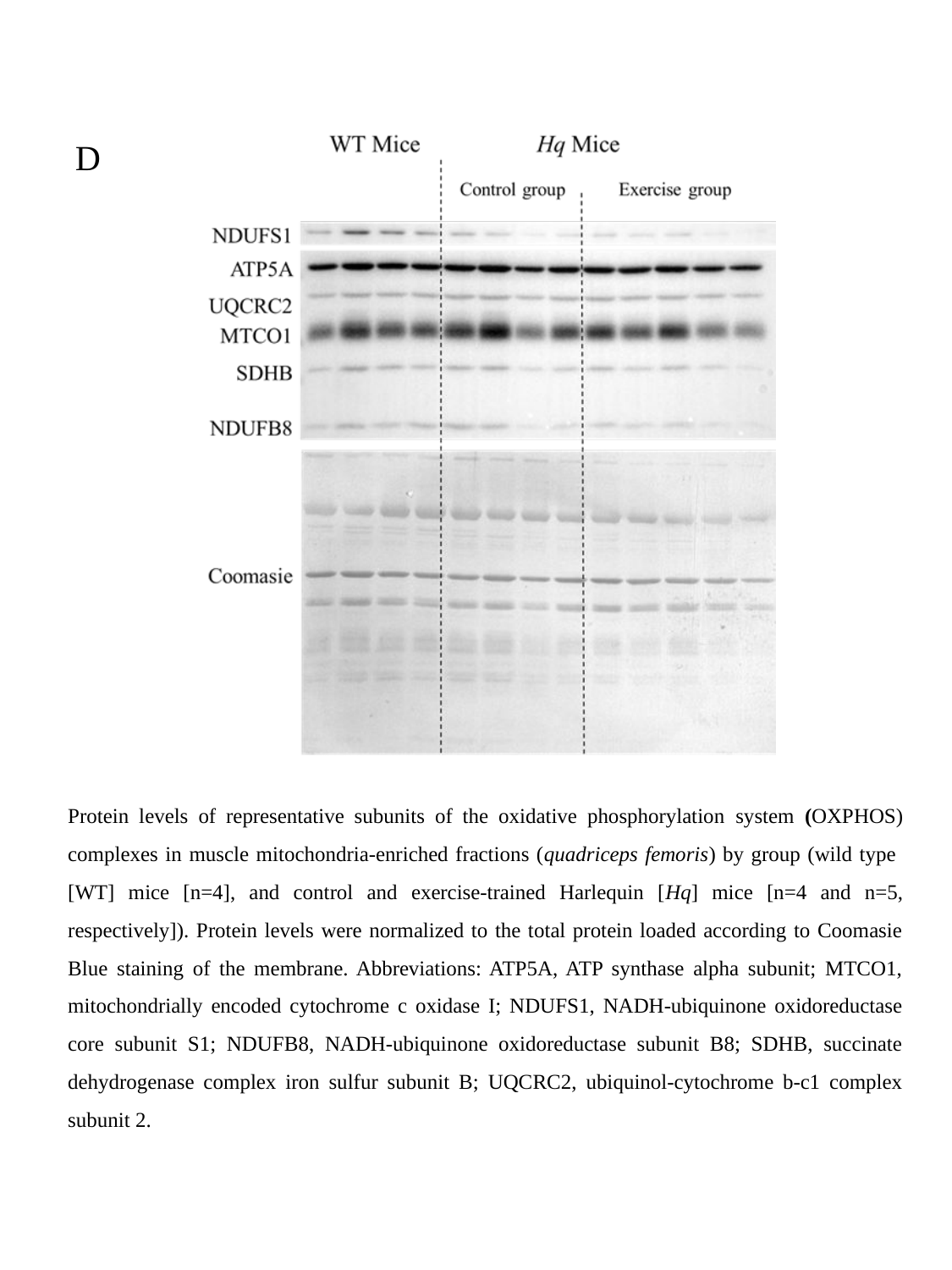

D
Protein levels of representative subunits of the oxidative phosphorylation system (OXPHOS) complexes in muscle mitochondria-enriched fractions (quadriceps femoris) by group (wild type [WT] mice [n=4], and control and exercise-trained Harlequin [Hq] mice [n=4 and n=5, respectively]). Protein levels were normalized to the total protein loaded according to Coomasie Blue staining of the membrane. Abbreviations: ATP5A, ATP synthase alpha subunit; MTCO1, mitochondrially encoded cytochrome c oxidase I; NDUFS1, NADH-ubiquinone oxidoreductase core subunit S1; NDUFB8, NADH-ubiquinone oxidoreductase subunit B8; SDHB, succinate dehydrogenase complex iron sulfur subunit B; UQCRC2, ubiquinol-cytochrome b-c1 complex subunit 2.

## Slide 7
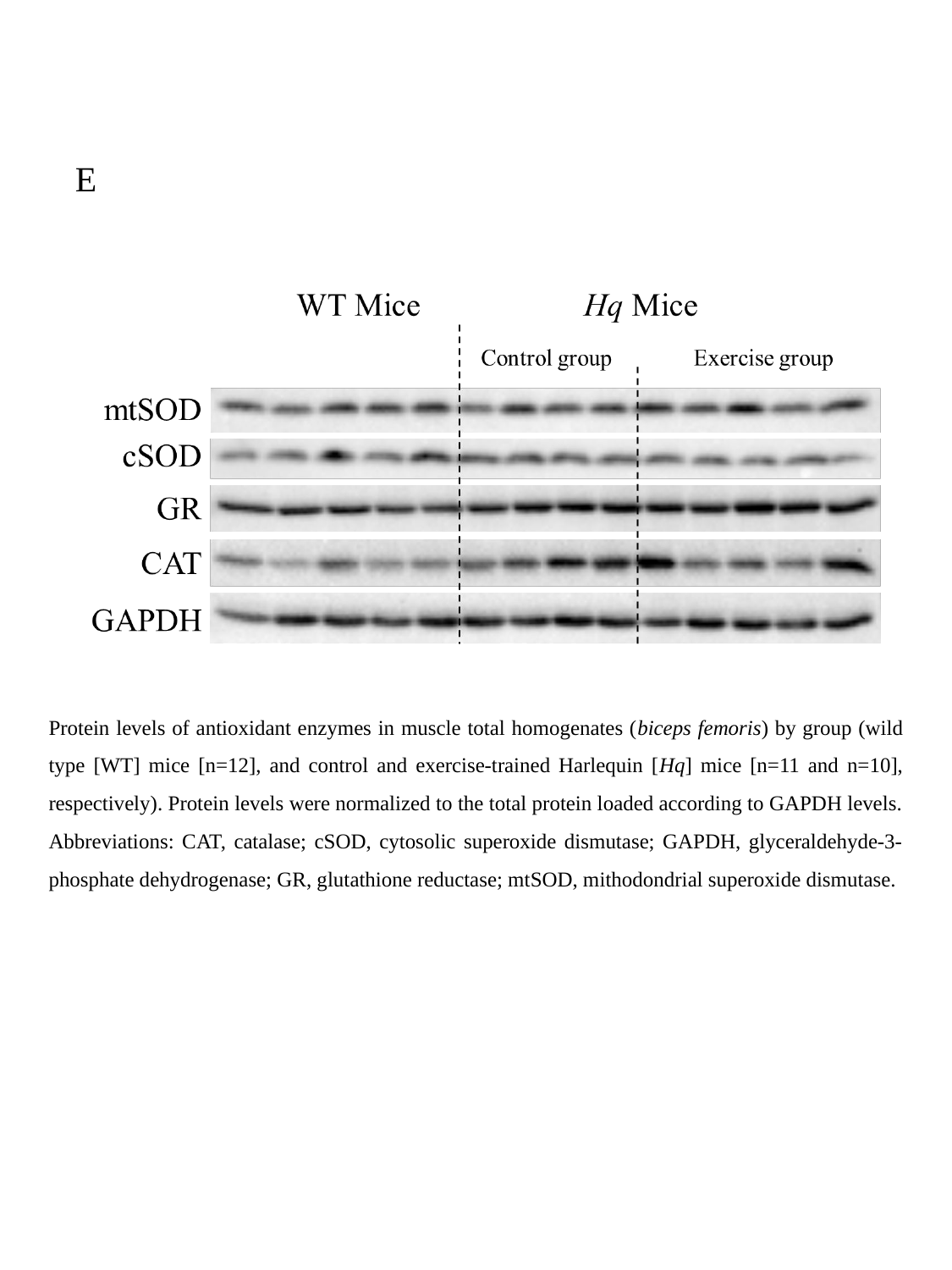

E
Protein levels of antioxidant enzymes in muscle total homogenates (biceps femoris) by group (wild type [WT] mice [n=12], and control and exercise-trained Harlequin [Hq] mice [n=11 and n=10], respectively). Protein levels were normalized to the total protein loaded according to GAPDH levels. Abbreviations: CAT, catalase; cSOD, cytosolic superoxide dismutase; GAPDH, glyceraldehyde-3-phosphate dehydrogenase; GR, glutathione reductase; mtSOD, mithodondrial superoxide dismutase.

## Slide 8
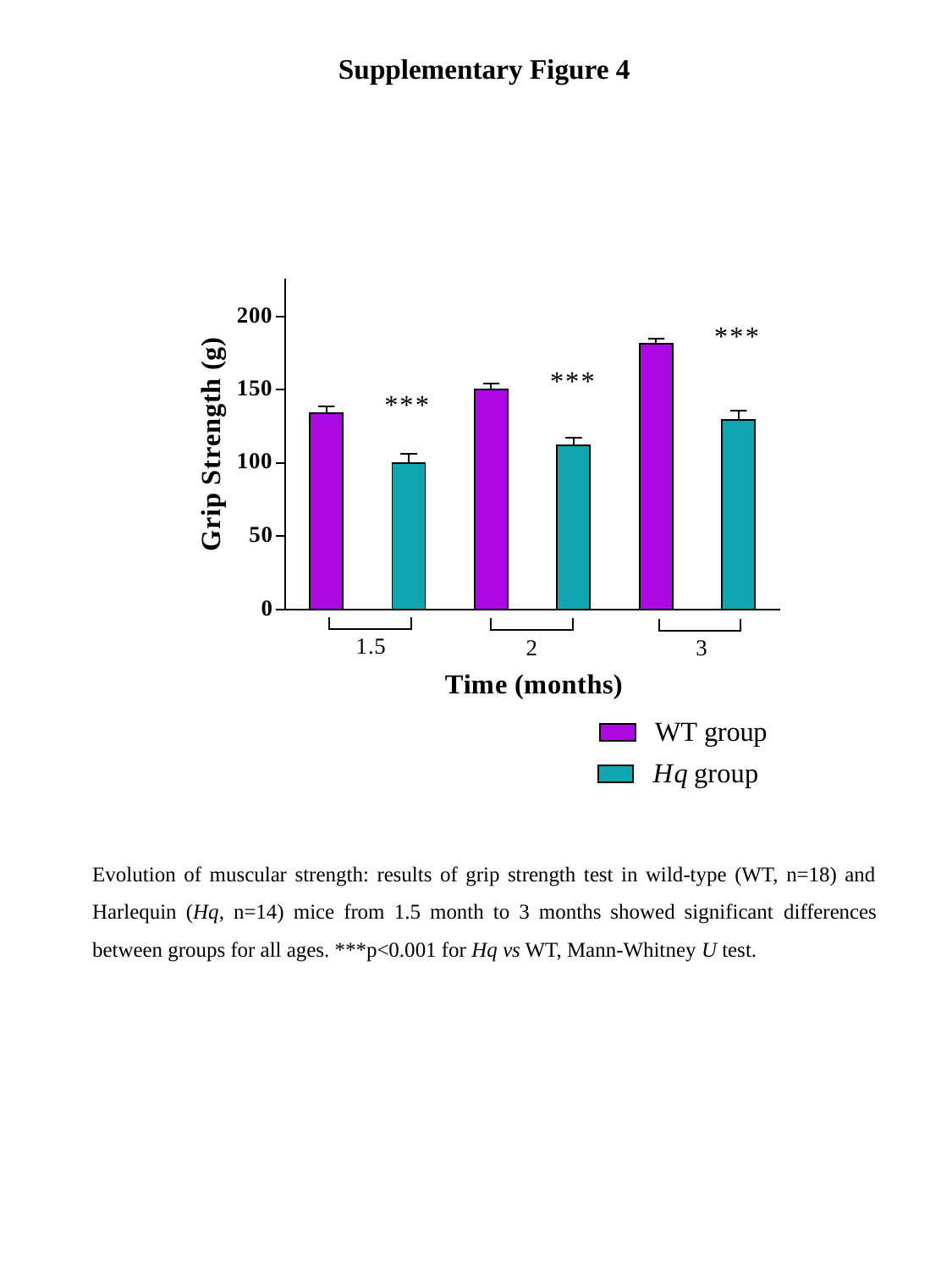

Supplementary Figure 4
Evolution of muscular strength: results of grip strength test in wild-type (WT, n=18) and Harlequin (Hq, n=14) mice from 1.5 month to 3 months showed significant differences between groups for all ages. ***p<0.001 for Hq vs WT, Mann-Whitney U test.

## Slide 9
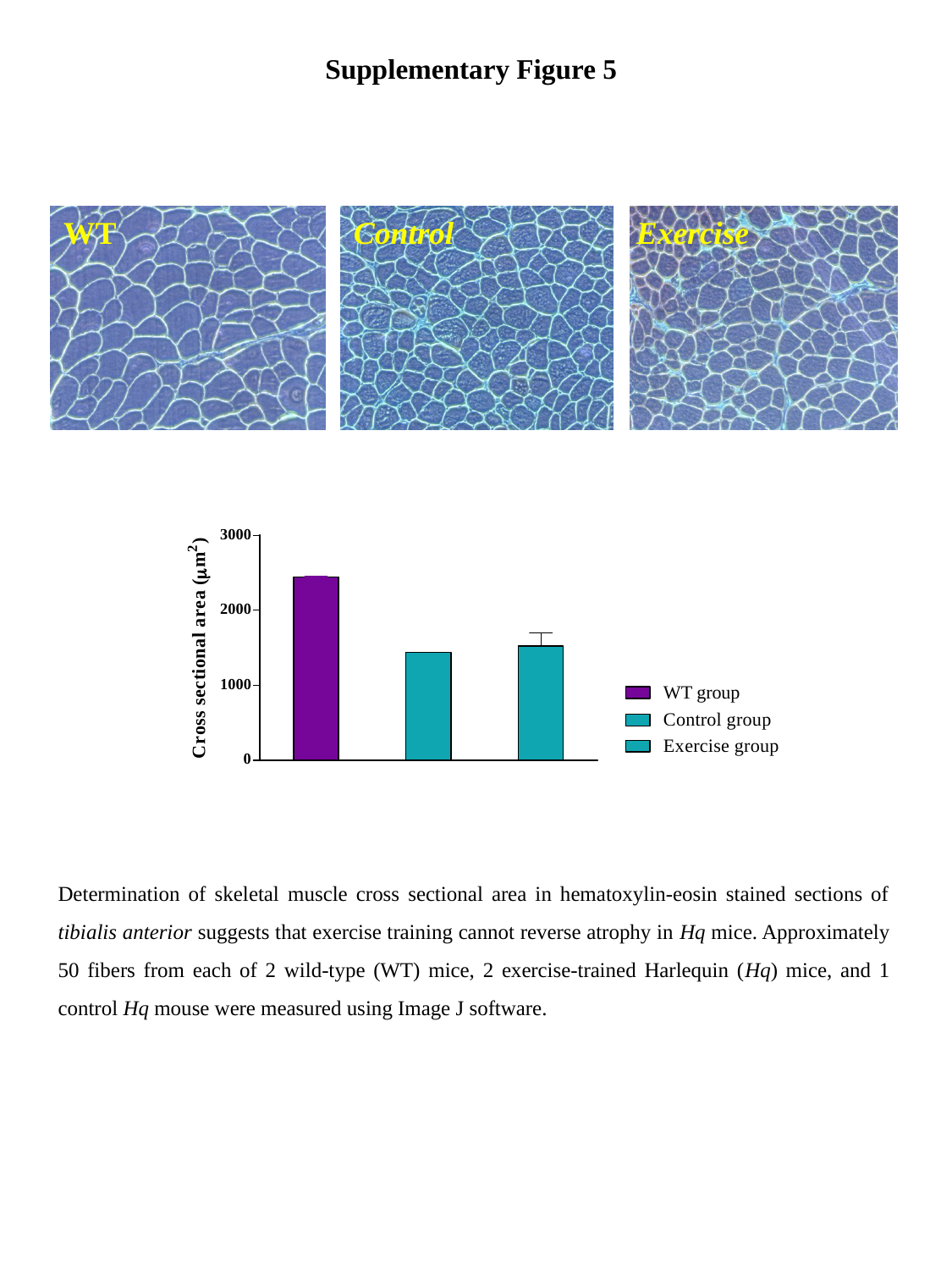

Supplementary Figure 5
WT
Control
Exercise
Determination of skeletal muscle cross sectional area in hematoxylin-eosin stained sections of tibialis anterior suggests that exercise training cannot reverse atrophy in Hq mice. Approximately 50 fibers from each of 2 wild-type (WT) mice, 2 exercise-trained Harlequin (Hq) mice, and 1 control Hq mouse were measured using Image J software.
